# ATP Binding Cassette Transporter A1 is Involved in Extracellular Secretion of Acetylated APE1/Ref-1

**DOI:** 10.3390/ijms20133178

**Published:** 2019-06-28

**Authors:** Yu Ran Lee, Hee Kyoung Joo, Eun Ok Lee, Hyun Sil Cho, Sunga Choi, Cuk-Seong Kim, Byeong Hwa Jeon

**Affiliations:** Research Institute of Medical Sciences, Department of Physiology, College of Medicine, Chungnam National University, 266 Munhwa-ro, Jung-gu, Daejeon 35015, Korea

**Keywords:** APE1/Ref-1, Acetylation, Secretion, Non-classical pathway, ABCA1 transporter

## Abstract

Acetylation of nuclear apurinic/apyrimidinic endonuclease-1/redox factor-1 (APE1/Ref-1) is associated with its extracellular secretion, despite the lack of an N-terminal protein secretion signal. In this study, we investigated plasma membrane targeting and translocation of APE1/Ref-1 in HEK293T cells with enhanced acetylation. While APE1/Ref-1 targeting was not affected by inhibition of the endoplasmic reticulum/Golgi-dependent secretion, its secretion was reduced by inhibitors of ATP-binding cassette (ABC) transporters, and siRNA-mediated down-regulation of ABC transporter A1. The association between APE1/Ref-1 and ABCA1 transporter was confirmed by proximal ligation assay and immunoprecipitation experiments. An APE1/Ref-1 construct with mutated acetylation sites (K6/K7R) showed reduced co-localization with ABC transporter A1. Exposure of trichostatin A (TSA) induced the acetylation of APE1/Ref-1, which translocated into membrane fraction. Taken together, acetylation of APE1/Ref-1 is considered to be necessary for its extracellular targeting via non-classical secretory pathway using the ABCA1 transporter.

## 1. Introduction

Cells can dynamically communicate with the extracellular environment via secretory molecules. The secreted molecules can act as para- or autocrine factors in cell signaling and gene expression [1,2]. The two major pathways for secreting proteins across the plasma membrane in eukaryotes are the classical (endoplasmic reticulum (ER)/Golgi-dependent) and non-classical (endoplasmic reticulum (ER)/Golgi-independent or unconventional) pathways. Identification of the non-classical pathway was based on the observation that communication with neighboring cells via extracellular secretion of signaling proteins can occur even in absence of a signal peptide, an N-terminal extension sequence, or the ER membrane translocation machinery. Signaling molecules that are secreted via the non-classical pathway include: fibroblast growth factor-2 (FGF-2), an angiogenesis stimulant essential for tumor growth and metastasis [3]; galectin-1, an inducer for tumor-mediated immune suppression [4]; and cytokine interleukin-1β (IL-1 β) from activated macrophages, a mediator of the inflammatory response involved in cell proliferation, differentiation, and apoptosis [5].

The apurinic/apyrimidinic endonuclease-1/redox factor-1 (APE1/Ref-1), a typical nuclear protein with a pleiotropic role in oxidative stress response, has also been found in the mitochondria of metabolically active and proliferative cells [6,7]. Recent studies have indicated that APE1/Ref-1, which is localized in the cytoplasm, is susceptible to post-translational modification (PTM), such as nitrosylation or acetylation [8,9]. Interestingly, APE1/Ref-1 protein secretion showed remarkable regulatory effects on inflammatory cytokine-stimulated cells both *in vitro* and *in vivo* [10,11,12]. Moreover, in different tumor cell types, the level of APE1/Ref-1 was abnormally high [13,14], suggesting its possible use as a cancer biomarker. The different subcellular distribution of APE1/Ref-1 and the evidence of its extracellular secretion together suggest that its distribution and translocation are controlled not only by stimulatory agents like chemicals or hormones but also by plasma membrane transporters. Although APE1/Ref-1 has been demonstrated to accumulate along the plasma membrane in the presence of a deacetylase inhibitor, trichostatin A (TSA) [15], the mechanism of its translocation across the plasma membrane remains unknown. 

In this study, we hypothesized that APE1/Ref-1 is secreted by a membrane transporter since its plasma membrane translocation depends on PTM [16,17,18], and appears to be controlled by extracellular factors whose identities are still under investigation. To identify the transporter pathway for APE1/Ref-1, we used pharmacological inhibitors that prevent the secretion of intracellular proteins by targeting specific transporters, such as the ABC transporter A1 (ABCA1). In the present study, we established the identity of the transport pathway for APE1/Ref-1 and our results will facilitate the analysis of the extracellular roles of this multifunctional protein.

## 2. Results

### 2.1. Non-Classical Secretion of APE1/Ref-1 in Response to TSA

**Figure 1:**
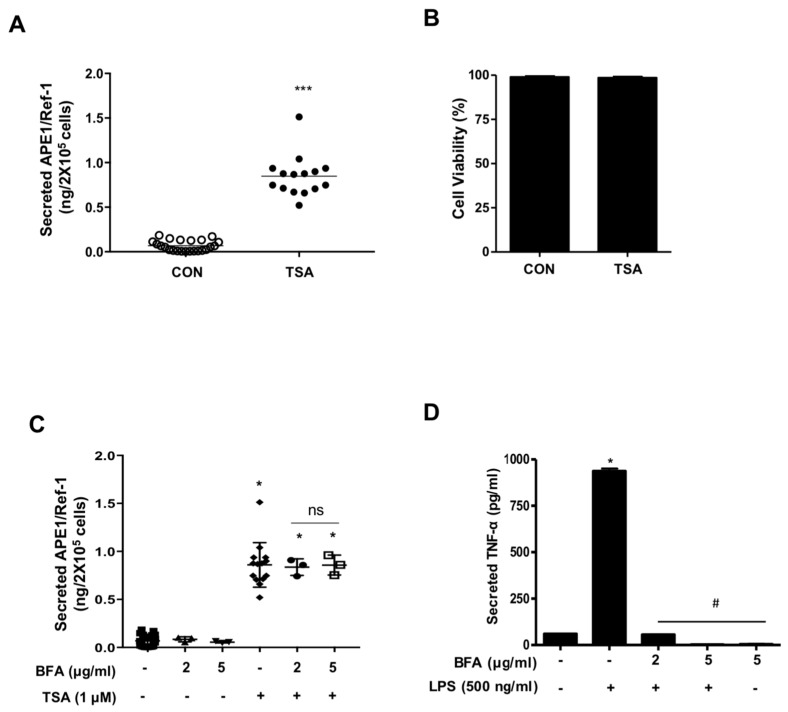
Secretion of APE1/Ref-1 was mediated by the non-classical pathway. (**A**) HEK293T cells were treated with 1 µM trichostatin A (TSA) for 1 h prior to collecting the cell-free supernatant. The total extracellular secretion of APE1/Ref-1 from acetylated HEK293T cells was measured by ELISA. *Columns*, mean (*n* = 15-20); *dot plot*, SE. *** *p* < 0.001 indicates a significantly different result from control cells according to an unpaired *t-*test. (**B**) Effects of TSA on the viability of HEK293T cells were determined using a bioluminescent assay *Columns*, mean (*n* = 6); *bars*, non- significantly different from control cells according to an unpaired *t-*test. (**C**) HEK293T cells were pretreated with brefeldin A (BFA), a classical secretory pathway inhibitor, followed by 1 µM TSA treatment for 3 h. The total amount of secreted Ac-APE1/Ref-1 in cell-free supernatant was analyzed by ELISA. (**D**) The total amount of secreted TNF-α from LPS-stimulated RAW 264.7 cells before exposure to BFA at the indicated concentration. *Column,* mean (*n* = 5); *bars*, SE. *^,#^, *p* < 0.05 indicates a significantly different result compared with control or between groups by one-way ANOVA followed by Bonferroni’s multiple comparison test.

Initially, we determined the secretion of APE1/Ref-1 in trichostatin A (TSA)-treated HEK293T cells. The amount of secreted APE1/Ref-1 in culture supernatant, in response to deacetylase inhibitor, was quantitatively analyzed by enzyme-linked immunosorbent assay (ELISA), and the cell viability was analyzed by RealTime-Glo^TM^ MT assay to exclude the possibility of contamination from cell cycle progression. The results are summarized in Figure 1A,B. TSA concentrations used in the present study were within the range used previously to document the acetylation of intracellular proteins [9], without affecting the viability of HEK293T cells (Figure 1B). Interestingly, TSA-treated cells showed an increase in APE1/Ref-1 secretion (total amount of APE1/Ref-1, 0.85 ± 0.06 ng/2 × 10^5^ cells) compared to the control cells (total amount of APE1/Ref-1, 0.07 ± 0.01 ng/2 × 10^5^ cells) (Figure 1A). Since TSA induced APE1/Ref-1 secretion, we examined the effects of secretory pathway inhibitors on its secretion. Pre-exposure of TSA-treated HEK293T cells to brefeldin A (BFA), an inhibitor of the ER-to-Golgi classical transport pathway, had no effect on Ac-APE1/Ref-1 secretion (Figure 1C), whereas TNF-α secretion, which depends on the classical secretory pathway, was abrogated (Figure 1D). These results together indicated that TSA treatment increased APE1/Ref-1 secretion via non-classical transport pathways.

### 2.2. Extracellular Secretion of APE1/Ref-1 Was Decreased by ABC Transporter Inhibitors

**Figure 2:**
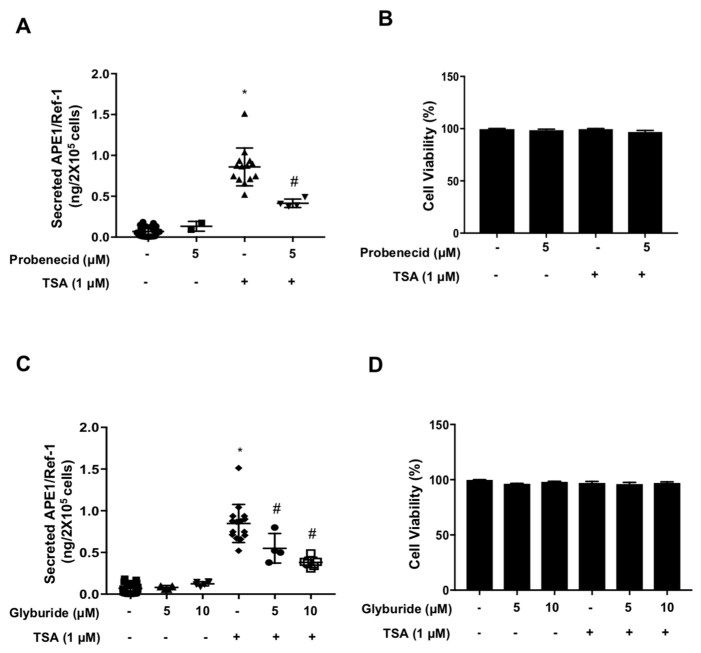
Secretion of APE1/Ref-1 was decreased by ABC transporter inhibitors. Quantitative analysis of Ac-APE1/Ref-1 secretion in the presence of pharmacological inhibitors. (**A**) Probenecid or (**C**) glyburide, non-classical secretory pathway inhibitors, for 1h at the indicated concentrations, followed by 1 µM TSA treatment for 3 h. *Column* mean (*n* = 4–5); *dot plot*, SE. ***, *p* < 0.001 indicates a significantly different result compared with control or between group by one-way ANOVA followed by Bonferroni’s multiple comparison test. The total amount of secreted Ac-APE1/Ref-1 in cell-free supernatant was analyzed by ELISA. (**B**,**D**) Effect of inhibitor and TSA on the viability of HEK293T cells were determined using a bioluminescent assay. *Column* mean (*n* = 4–5); *bars*, SE. *^,#^, *p* < 0.05 indicates a significantly result different compared with control or between groups by one-way ANOVA followed by Bonferroni’s multiple comparison test.

ABC transporters were used to transport a variety of substrates across cell membrane including ions, lipid, amino acids, even proteins [19]. As established APE1/Ref-1 secretion is mediated with non-classical transport pathway, we focused on the effect of an ABC transporter on APE1/Ref-1 secretion in TSA-treated HEK293T cells. As shown in Figure 2A, the secretion of APE1/Ref-1 from TSA-treated HEK293T cells was affected when the cells were pre-exposed to probenecid, a broad-spectrum inhibitor of ABC transporters. Probenecid-pretreated cells stimulated with TSA (0.39 ± 0.02 ng/2 × 10 ^5^ cells) showed significantly inhibited APE1/Ref-1 secretion compared to TSA-only-stimulated cells (0.85 ± 0.06 ng/2 × 10 ^5^ cells). As shown in Figure 2C, interestingly, APE1/Ref-1 secretion was also significantly reduced by more than 50% after pretreatment with glyburide [20,21], a selective inhibitor of ABC transporters such as ABCA1, ABCB1, ABCB11, ABCC1, ABCC2, ABCC3, ABCC8, and ABCC9 [22,23,24,25,26,27]. The exposure of two inhibitors did not affect cell viability, as shown in Figure 2B,D, respectively. These results strongly suggested that secretion of APE1/Ref-1 in TSA-treated HEK293T cells depends on the non-classical secretory pathway involving ABC transporters.

### 2.3. ABCA1 Transporter Was Involved in the Secretion of APE1/Ref-1 

**Figure 3:**
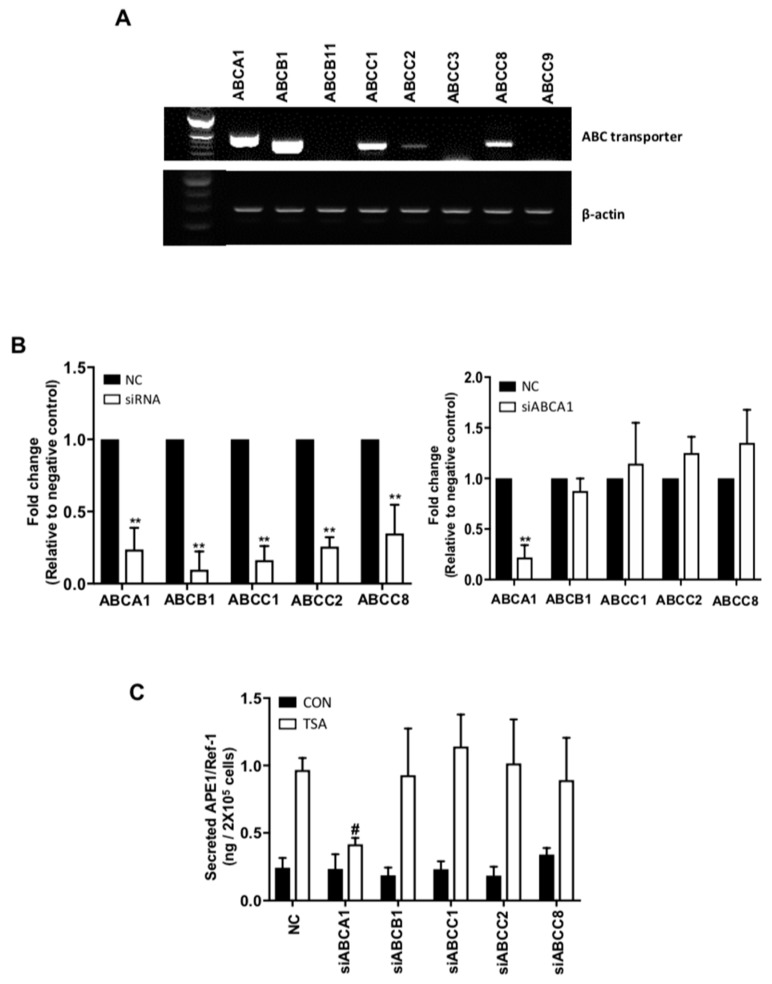
ATP binding cassette transporter A1 (ABCA1) was involved in the secretion of APE1/Ref-1. (**A**) The subfamily of ABC transporter expressed in HEK293T cells. The qRT-PCR analysis for ABC transporters using lysates from HEK293T cells. β-actin was tested in parallel and normalized an equal amounts of total RNA per sample. (**B**) The subfamily of ABC transporter expressed in HEK293T cells transiently transfected with siRNA. (Left) The qRT-PCR analysis for knock-down of ABC transporters in transfected cells with each siRNA. (Right) mRNA level of each ABC transporters were analyzed using qRT-PCR in siABCA1 transfected cells. *Columns*, mean (*n* = 3); *bars*, SE. ** *p* < 0.01 indicated a significantly different result from non-specific siRNA (NC) transfected cells by two-way ANOVA followed by Bonferroni’s multiple comparison test. (**C**) Changes in the total amount of secreted Ac-APE1/Ref-1 from cells after knock-down of each ABC transporter were analyzed using ELISA. *Columns*, mean (*n* = 3); *bars*, SE. ^#^ *p* < 0.05 indicated a significantly different result from TSA treated cells or between groups by two-way ANOVA followed by Bonferroni’s multiple comparison test.

To identify the ABC transporter responsible for the secretion of APE1/Ref-1, we determined the presence of ABC transporters in HEK293T cells. Expression of ABCA1, ABCB1, ABCC1, ABCC2, and ABCC8 transporters were detected in the HEK293T cells (Figure 3A). We proceeded to determine the functional significance of each ABC transporter in TSA-treated HEK293T cells using siRNA. Transient transfection of each ABC transporter-targeted siRNA resulted in selective and significant abrogation of the respective ABC transporter (Figure 3B left panel). Importantly, specific knockdown of ABCA1 without affecting the expression of other subtypes (Figure 3B right panel), caused significant inhibition of APE1/Ref-1 secretion in HEK293T cells, despite the TSA treatment; from 0.96 ng/2 × 10^5^ cells in TSA-treated cells to 0.42 ng/2 × 10^5^ cells in ABCA1 knockdown TSA-treated cells (Figure 3C). The latter amount was comparable to values obtained in other control cells. Knockdown of other ABC transporters did not significantly affect APE1/Ref-1 secretion. In summary, these results indicated that the ABCA1 transporter was mainly required for the extracellular secretion of APE1/Ref-1 in TSA-treated HEK293T cells.

### 2.4. Acetylation of APE1/Ref-1 Was Required for Binding to ABCA1 Transporter

**Figure 4:**
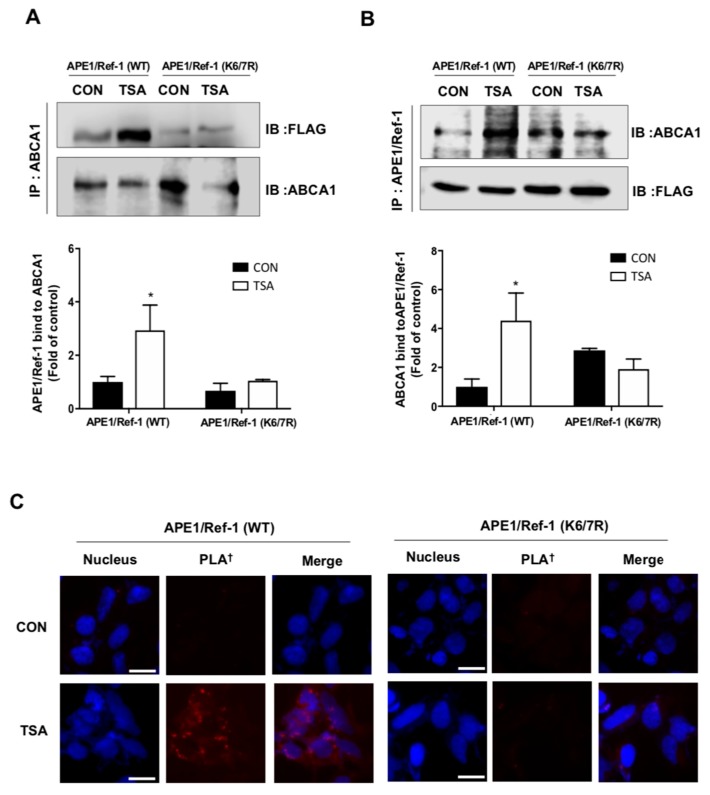
Plasma membrane associated APE1/Ref-1 is bound to ABCA1 in response to acetylation. Cells transiently expressing wild type APE1/Ref-1-FLAG or mutant APE1/Ref-1(K6/7R)-FLAG were treated with 1 µM TSA for 1 h. (**A**) Whole cell lysates were immunoprecipitated using the monoclonal anti-ABCA1 antibody, followed by immunoblot with the anti-FLAG antibody. (**B**) For reverse immunoprecipitation, cell lysates were immunoprecipitated with anti-APE1/Ref-1 antibody followed by immunoblot analysis with the polyclonal anti-ABCA1 antibody. Blots were stripped and re-probed with anti-ABCA1 or FLAG antibodies to ensure equal protein loading and no contamination of cellular proteins. Similar results were observed in replicate experiments. *Columns*, mean (*n* = 2-3); *bars*, SE. *, *p* < 0.05 indicates a significantly different result from control cells according to unpaired *t-*tests. (**C**) The binding between APE1/Ref-1 and ABCA1 in the plasma membrane was visualized using with a Duolink II PLA system with primary polyclonal anti-APE1/Ref-1 and monoclonal anti-ABCA1 antibodies (PLA^†^). The PLA-specific fluorescence which represents the APE1/Ref-1-ABCA1 signal, and the DAPI nuclear staining are in red and blue, respectively. The experiment was repeated multiple times with similar results; the data shown here are from a representative experiment. Optical slices were examined using a 40× oil immersion objective with a 2× zoom factor. Scale bar, 20 µm (×80).

We next tested whether the acetylation of APE1/Ref-1 was specifically required for both the binding to ABCA1 transporter and subsequent translocation of the protein to the exterior of the cell. We examined the role of acetylation using two different, yet complementary approaches. We first performed a co-immunoprecipitation experiment to confirm that APE1/Ref-1 was directly associated with the ABCA1 transporter. Acetylation of intracellular proteins by TSA treatment enabled the binding of exogenously overexpressed APE1/Ref-1-FLAG to the ABCA1 transporter, as demonstrated by the pull-down of FLAG-tagged APE1/Ref-1 protein using anti-ABCA1 transporter antibody. However, a less acetylated mutant form, the exogenously overexpressed APE1/Ref-1 (K6/7R)-FLAG, was not co-immunoprecipitated using anti-ABCA1 transporter antibody despite the stimulation of acetylation after TSA treatment (Figure 4A). Conversely, the binding of APE1/Ref-1 to ABCA1 was also confirmed by co-immunoprecipitation with anti-APE1/Ref-1 antibody; we observed that acetylation of APE1/Ref-1 was required for the interaction with ABCA1 (Figure 4B). The Duolink II cell-based fluorescent proximal ligation assay (PLA) was used to visualize the direct binding of APE1/Ref-1 to ABCA1. TSA-treated HEK293T cells, expressing wild- type APE1/Ref-1-FLAG, were incubated with a mixture of anti-APE1/Ref-1 and anti-ABCA1 antibodies. The association of APE1/Ref-1 with ABCA1 was visualized by numerous red spots, indicating a proximal interaction between APE1/Ref-1 and ABCA1 transporter (< 40 nm). In contrast, TSA-treated HEK293T cells, expressing mutant APE1/Ref-1(K6/7R)-FLAG, showed only weak background signals. These results demonstrated that APE1/Ref-1 directly interacted with ABCA1 transporter as a prerequisite for its secretion in TSA-treated HEK293T cells (Figure 4C). This result was also supported by comparing the efficient co-immunoprecipitation of wild-type APE1/Ref-1-FLAG with ABCA1.

### 2.5. Intracellular Acetylation Induced APE1/Ref-1 Translocation to the Plasma Membrane 

**Figure 5:**
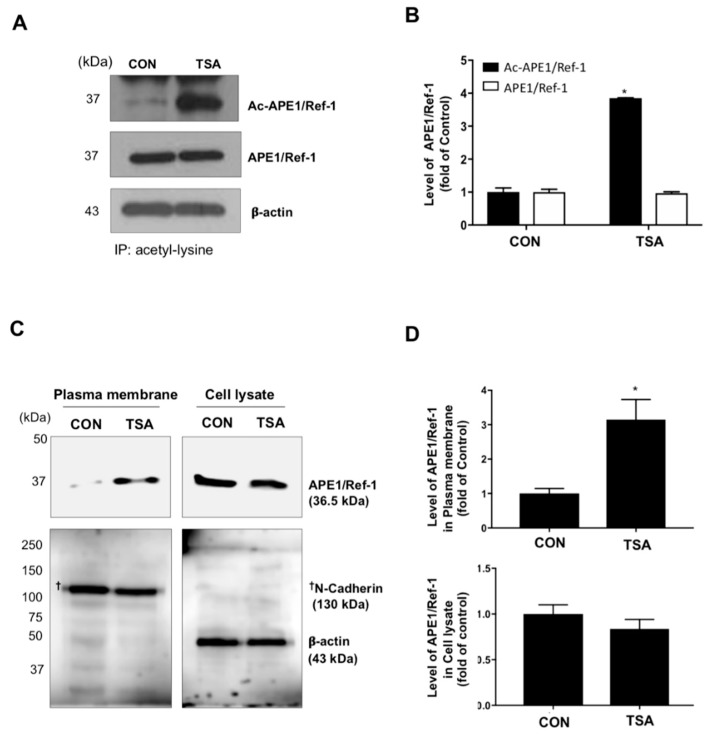
Intracellular APE1/Ref-1 was targeted to the plasma membrane in response to acetylation. (**A** and **B**) Whole cell lysates were immunoprecipitated using an anti-acetyl lysine antibody, followed by immunoblotting with the polyclonal anti-APE1/Ref-1 antibody. The blots were stripped and re-probed with anti-β-actin and APE1/Ref-1 antibody to ensure equal protein loading. Similar results were obtained from replicate experiments. *Column,* mean (*n* = 3); *bars*, SE. *, *p* < 0.05, significantly different compared with control or between group by one-way ANOVA followed by Bonferroni’s multiple comparison test. (**C**,**D**) Membrane fractions or whole cell lysates were prepared from the TSA-treated cells. Immunoblotting for APE1/Ref-1 was performed using the polyclonal anti-APE1/Ref-1 antibody. Blots were stripped and re-probed with anti-N-cadherin and anti-β-actin antibodies to control for differences in protein loading. Fold changes in the levels of APE1/Ref-1 in the plasma membrane fraction relative to the control are shown. ^†^, indicates molecular marker (N-cadherin) of left image. *Column* mean (*n* = 3); *bars*, SE.*, *p* < 0.05 indicates a significantly different result compared with control or between groups by one-way ANOVA followed by Bonferroni’s multiple comparison test.

Next, we examined whether APE1/Ref-1 can be acetylated with TSA, and the trafficking of acetylated APE1/Ref-1(Ac-APE1/Ref-1) to the plasma membrane in HEK293T cells. Acetylation of APE1/Ref-1 in the response of TSA was analyzed with immunoblotting of APE1/Ref-1 after immunoprecipitation using anti-acetyl-lysine antibody (Figure 5A). TSA-treated cells showed about 4-fold higher quantities of Ac-APE1/Ref-1, compared with control cells (Figure 5B). Finally, we proceeded to examine the trafficking of APE1/Ref-1 to the plasma membrane in TSA-treated HEK293T cells. As shown in Figure 5C, TSA-induced APE1/Ref-1 enrichment was detected in the plasma membrane fraction and was confirmed using an anti-N-cadherin antibody. TSA-treated cells had an approximately 3-fold higher content of plasma membrane-located APE1/Ref-1 compared to the control cells (Figure 5D). 

## 3. Discussion

Previous studies have demonstrated that APE1/Ref-1, a typical nuclear protein, is also present in the extracellular milieu of cells associated with chronic diseases [11,13,28,29,30]. Identification of its secretory pathway would clarify whether the detection of APE1/Ref-1 in extracellular samples is an experimental artifact or is the result of a controlled cellular response to certain stimulators. The major finding of this study was that APE1/Ref-1 is secreted from cells stimulated for protein acetylation via a non-classical secretory pathway that involves ABCA1 transporter. Since probenecid and glyburide treatment caused significant inhibition of Ac-APE1/Ref-1 secretion, suggesting the involvement of a non-classical secretory pathway with ABCA1 transporter, we focused on ABCA1 as a transporter candidate based on the following experimental data: (i) dominant expression in HEK293T cells, (ii) transporter-specific pharmacological inhibition, and (iii) knock-down using specific ABCA1 siRNA. These observations strongly supported the possibility of involvement of ABCA1 in the secretion of Ac-APE1/Ref-1. 

Some studies have suggested that protein translocation mediated by ABC transporters requires protein unfolding, which is a general prerequisite for protein translocation complexes across membranes [31,32]. However, some non-classical secretion of FGF-2 and Gal-1 does not require protein unfolding [33,34]. ABCA1 is a membrane protein, and affects secretion of various proteins such as the macrophage migration inhibitory factor (MIF) and interleukin-1beta (IL-1β) [20,26]. Moreover, the direct involvement of ABC transporters has been demonstrated for lipid transport [35], such as vesicle export [36]. In a previous study, TSA-mediated acetylation was shown to cause PTM of APE1/Ref-1 (30 Lys residues in APE1/Ref-1), attaching an acetyl group to the ε-amino group of lysine residues [37]. Acetylation reduces the net charge and increases the hydrophobicity of APE1/Ref-1 [38]. In the present study, APE1/Ref-1 secretion was not limited with HDAC inhibitor TSA. HDAC inhibitors such as SAHA, valproic acid, and butyrate also induced APE1/Ref-1 secretion which was inhibited by glyburide (Supplementary Figure S1). Pharmacological inhibitions of probenecid and glyburide on APE1/Ref-1 secretion suggested a possible involvement of ABC transporters. Sulfonylurea such as glyburide used for the treatment of diabetes mellitus inhibits a variety of ABC transporters, including ABCA1 [22,23,24,25,26,27], the specificity of glyburide for each ABC transporters should be carefully considered. In the present study, we have confirmed that the expression of ABCA1, ABCB1, ABCC1, ABCC2, and ABCC8 transporters were detected in the HEK293T cells. Specific knockdown of ABCA1 without affecting the expression of other ABC transporters caused significant inhibition of APE1/Ref-1 secretion in HEK293T cells. Our study is the first to describe the extracellular secretion of Ac-APE1/Ref-1 by ABCA1 in response to acetylation. However, it is still unknown how ABCA1 transports APE1/Ref-1. It is necessary to further research APE1/Ref-1 secretion in response to specific stimuli such as an endogenous hormone. 

Intracellular imbalance can influence the interplay between survival pathways and cell death, causing cell cycle arrest, re-differentiation, or metabolic reprogramming. Intracellular imbalance is increased under inflammatory conditions associated with lung disease [39], vascular disease [40], hemorrhage, and sepsis [41], acetylation combined with coordinated expression of multiple inflammatory genes. Considering these reports, TSA-mediated acetylation in this study is an important observation for explaining the export of intracellular APE1/Ref-1. Recently, we have shown that APE1/Ref-1 is extracellularly secreted in an LPS-stimulated endotoxemic rat model [11]. The extracellular APE1/Ref-1 plays the role in disulfide bond exchange within the TNF receptor, causing inhibition of vascular inflammatory signals [10]. Stimulation of the APE1/Ref-1 secretion is triggered by its acetylation, as shown by diminished secretion level of the mutant APE1/Ref-1(K6/7R) due to mutated lysine, thus supporting the critical role played by lysine acetylation in various cardiovascular diseases [28]. Although deacetylation of Ac-APE1/Ref-1 followed time-dependent kinetics, eventually reaching an optimum, the recovery mechanism of Ac-APE1/Ref-1 to its non-acetylated form with respect to its reductase activity requires further studies. These observations raised the question of whether secreted APE1/Ref-1 can be influenced in an extracellular inflammatory environment. These results also suggested that extracellular APE1/Ref-1 with redox activity could be considered for further clinical investigation to determine its possible therapeutic efficacy against chronic inflammation, owing to its potential role in the early prevention of inflammatory signals *in vivo*. 

In summary, our current study indicated that the extracellular secretion of APE1/Ref-1 depends on acetylation, a common PTM in eukaryotic cells. Ac-APE1/Ref-1 is intracellularly targeted to the plasma membrane and translocated by ABCA1 membrane transporter for secretion into the extracellular milieu.

## 4. Materials and Methods

### 4.1. Materials

TSA, brefeldin A (BFA), glyburide, probenecid, suberoylanilide hydroxamic acid (SAHA), valproic acid, butyrate and lipopolysaccharide (LPS) were purchased from Sigma Aldrich (St. Louis, MO, USA). Lipofectamine® RNAiMAX, Opti-MEM™ media, and Dynabeads^®^ Magnetic beads were purchased from Thermo Fisher Scientific (San Jose, CA, USA). ABCA1 small interfering RNA (siRNA), control siRNA, and primers were purchased from Bioneer (Daejeon, Korea). Complementary DNA synthesis kit and PCR PreMix kit were purchased from Intron biotechnology Inc. (Seongnam, Gyeonggi-do, Korea). Mouse tumor necrosis factor (TNF)-α ELISA kit was purchased from R&D system (Minneapolis, MN, USA). Anti-N-cadherin antibody was purchased from Abcam (cat.no. ab18203, Cambridge, MA, USA); anti-β-actin antibody was purchased from Sigma Aldrich (cat.no. A5316, St. Louis, MO, USA); Monoclonal anti-ABCA1 antibody (cat.no. sc-58219, Santa Cruz Biotechnology, Santa Cruz, CA, USA) and polyclonal antibody (cat.no. PA1-16789, Invitrogen, Waltham, MA, USA) were used. Monoclonal (cat.no. NB100-116, Novus Biologicals, Littleton, CO, USA) and polyclonal antibodies against APE1/Ref-1 (cat.no. MR-PAAPE, MediRedox, Daejeon, Korea) were used. 

### 4.2. Cell culture and treatment 

Human embryonic kidney 293T (HEK293T) cells and Raw 264.7 cells were cultured in Dulbecco’s modified Eagle’s medium (Welgen, Korea) with 10% fetal bovine serum and 1% antibiotics. Each cell line was incubated under humid conditions at 37°C and 5% CO_2_. Cells were seeded into well plates and incubated for 24 hours, followed by replacement of the culture medium with Opti-MEM™. The cells were treated with the inhibitor for 2 h, followed by TSA treatment for 1 h, as indicated. Cell viability was analyzed using the RealTime-Glo^TM^ MT luminescent kit (Promega, Madison, WI, USA) in an opaque-walled assay plate, according to the manufacturer’s instructions and as reported previously [42].

### 4.3. Enzyme-linked immunosorbent assay (ELISA)

The amount of APE1/Ref-1 in each sample was determined by ELISA analysis (MediRedox, Daejeon, Korea) [43]. HEK293T cells were grown to confluence on a 12-well plate (2 × 10^5^ cells/well) and then changed with Opti-MEM™ medium. Then, the cells were treated with 1 μM TSA in 0.5 mL Opti-MEM™/well, and to obtain cell-free supernatant, the medium was collected by centrifugation at 1200 rpm for 3 min. Supernatant without cell debris was carefully recovered and re-centrifuged at 3000 rpm for 3 min. The final supernatant was used for detection of secretory APE1/Ref-1. Each sample was assayed in duplicate. 

For the measurement of TNF-α concentrations in the cell culture medium, the supernatant was obtained from LPS (300 ng/ml)-treated Raw 264.7 cells. The level of TNF-α was determined using a mouse TNF-α ELISA kit (BD Pharmingen, San Diego, CA, USA).

### 4.4. Transfection of small interfering RNA (siRNA)

To evaluate the efficiency of ABC transporters gene silencing, HEK293T cells (2.5 × 10 ^5^ cells/well) were transfected with a range of 20 nM ABC transporters siRNA (Bioneer Inc., Daejeon, Korea) or 20 nM control siRNA using Lipofectamine^®^ RNAiMAX according to the manufacturer’s protocol. The siRNA sequence of ABC transporters is shown in Table 1. 

### 4.5. Quantitative Real-Time Reverse Transcription-Polymerase Chain Reaction (qRT-PCR)

Total RNA from the HEK293T cells was extracted using NucleoSpin RNA Plus (Machery-Nagel Inc, Bethlehem, PA, USA) following manufacturer’s protocol. Complementary DNA (cDNA) was synthesized by reverse transcription-PCR kit (iNtRON Biotechnology, Gyeonggi-do, Korea) with primers [44]. The primers for human ABC transporters used in this study are shown in Table 2. The mRNA levels of ABC transporters were determined by qRT-PCR using SYBR Green PCR Master Mix (Promega, Madison, WI, USA) according to the protocols provided by the manufacturer with an ABI prism 7700 Sequence Detector System (Applied Biosystems, CA, USA). Target gene mRNA expression levels were calculated using the Δ*C*t method and normalized to glyceraldehyde 3-phosphate dehydrogenase mRNA expression.

### 4.6. Fractionation of Plasma Membrane 

The plasma membrane of HEK293T cells, which had been transfected with ABCA1 siRNA, was prepared by sucrose density gradient centrifugation as described previously [15]. The membrane pellet was analyzed by immunoblotting using anti-ABCA1 and anti-N-cadherin antibodies.

### 4.7. Immunoprecipitation

To analyze the Ac-APE1/Ref-1 in cell lysate, immunoprecipitation using anti-acetyl lysine antibody was performed as previously reported [15] with some modifications. Co-immunoprecipitation using monoclonal anti-ABCA1 antibody and polyclonal anti-APE1/Ref-1 was also performed to analyze the binding between ABCA1 and APE1/Ref-1 in cell lysate. Briefly, the one microgram of each antibodies was added to cell lysate and incubated for 2 h at 4 °C. Protein A/G agarose beads were then added to each sample and incubated for 18 h at 4 °C. The immunocomplex was collected using centrifugation at 300 rpm for 3 min and washed three times in washing buffer. Each sample was subjected to 10~12% SDS-PAGE, which was followed by immunoblotting using monoclonal anti-APE1/Ref-1 antibody and polyclonal anti-ABCA1 antibody.

### 4.8. Proximal Ligation Assay

Interaction of ABCA1 and APE1/Ref-1 was visualized using a Duolink II fluorescence kit (Sigma-Aldrich, St Louis, MO, USA) as described previously [15], with some modifications. HEK293T cells expressing wild type APE1/Ref-1-FLAG [45] or mutant APE1/Ref-1(K6/7R)-FLAG [46] grown on glass coverslips were treated with TSA for 1h. After fixation and blocking the cells were incubated with a mixture of goat anti-ABCA1 antibody (1:200) and rabbit anti-APE1/Ref-1 antibody (1:500) overnight at 4 °C. For *in situ* analysis, conjugated oligonucleotides [PLA probe anti-goat minus (minus strand) plus anti-rabbit antibody (plus strand)] were added and incubated for 1 h at 37 °C. A negative control was prepared by adding anti-mouse IgG. Fluorescence confocal microscopic images of the cells were acquired using excitation/emission wavelengths of 590/670 nm for the PLA signal and 410/470 nm for the DAPI (Sigma-Aldrich, St Louis, MO, USA). Images were acquired with a confocal TCS SP8 X microscope (Leica, Wetzlar, Germany) with 40× oil immersion objective. Z-stack image were collected at 0.4 µm intervals. The obtained images were processed using Leica LAS X software (Leica, Wetzlar, Germany).

### 4.9. Statistical analysis 

Values are expressed as means ± standard error of means (SEM). Statistical significance of differences in measured variables between control and treated groups was determined by paired *t*-test, one-way ANOVA or two-way ANOVA followed by Bonferroni’s multiple comparison tests. The differences were considered to be significant at *p* < 0.05.

## Figures and Tables

**Table 1:** The siRNA sequences of ABC transporters.

Target Gene	Accession No.	siRNA sequence (5′–3′)
*ABCA1*	NM_005502	GUGUCUAUAUGCAACAGAU
*ABCB1*	NM_000927	CAGCAAUUAGAACUGUGAU
*ABCC1*	NM_004996	CUGACAAGCUAGACCAUGA
*ABCC2*	NM_000392	ACAAGGUAAUGGUCCUAGA
*ABCC8*	NM_000352	CGUCAUCUCCUAUGUCACA

**Table 2:** The primers for glyburide-sensitive human ABC transporters.

Target Gene	Accession No.	Forward (5′–3′)	Reverse (5′–3′)	TM (℃)	Cycle
*ABCA1*	NM_005502	actgtggacaagatgctgagg	aaacatcacctcctgtcgcat	60	25
*ABCB1*	NM_000927	cacaagcccaagacagaaagc	tgagcatggatcggaaaacca	60	25
*ABCB11*	NM_003742	gaagctgagcctggtcatctt	gcagagatcaccctgaacaca	60	25
*ABCC1*	NM_004996	tgctcactttctggctggtag	ccgacgtgtcctccttgttta	60	25
*ABCC2*	NM_000392	tgcggctctcattcagtcttt	atcaccccaacacctgctaag	60	25
*ABCC3*	NM_003786	cgtggctacatcatcctctcc	gtagaaggtggtgaagcggaa	60	25
*ABCC8*	NM_000352	atccccacactgtccaacatc	agagagcaggcttcaatgacc	60	25
*ABCC9*	NM_020297	caggacggattatctgggagc	tgatgtaagccttgacgtgct	60	25

## Supplementary Materials

Supplementary materials can be found at https://www.mdpi.com/1422-0067/20/13/3178/s1. **Figure S1:** HDAC inhibitors induced APE1/Ref-1 secretion in HEK293T cells. (A) Effect of HDAC inhibitors on APE1/Ref-1 secretion in HEK293T cells. Cells (2 x 105 cells) were pretreated with trichostatin A (TSA, 1µM), SAHA (0.5 µM), valproic acid (1 mM), butyrate (0.5 mM) for 1h, then glyburide (10 µM), an ABCA1 transporter inhibitor was treated for 1 h. The total amount of secreted APE1/Ref-1 in cell-free supernatant was analyzed by ELISA as described in Material and Methods. Column mean (n = 4); bars, SE.* P < 0.05, significantly different compared with control. # P < 0.05, significantly different compared with Basal and glyburide group by one-way ANOVA followed by Bonferroni’s multiple comparison test. (B) Effect of glyburide and HDAC inhibitors on the viability of HEK293T cells were determined using a RealTime-GloTM MT luminescent kit. Note: HDAC inhibitors [TSA (1µM), SAHA (0.5 µM), valproic acid (1 mM), butyrate (0.5 mM)] used did not significantly affect cell viability.
