# Supplementary material for: ATP Binding Cassette Transporter A1 is Involved in Extracellular Secretion of Acetylated APE1/Ref-1
Source: Int J Mol Sci. 2019 Jun 28;20(13):3178. doi: 10.3390/ijms20133178 (PMC6651529; doi:10.3390/ijms20133178)
Supplement: Supplementary file 1 [file ijms-20-03178-s001.pdf]

# Supplementary figure

A

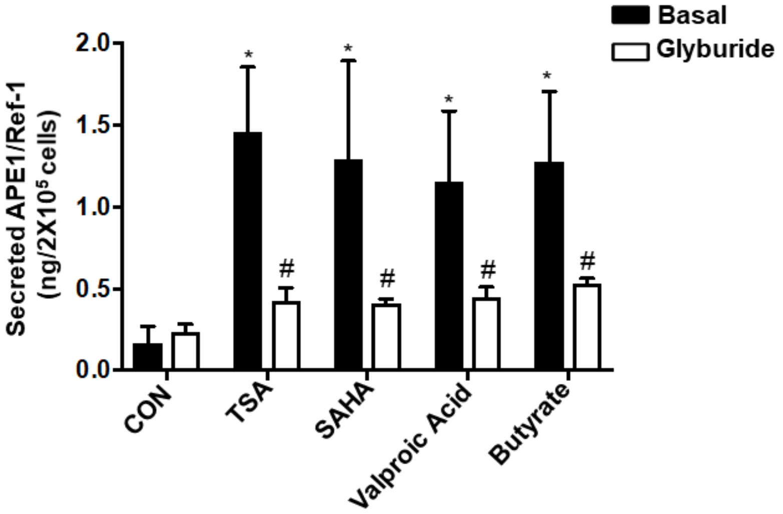


B

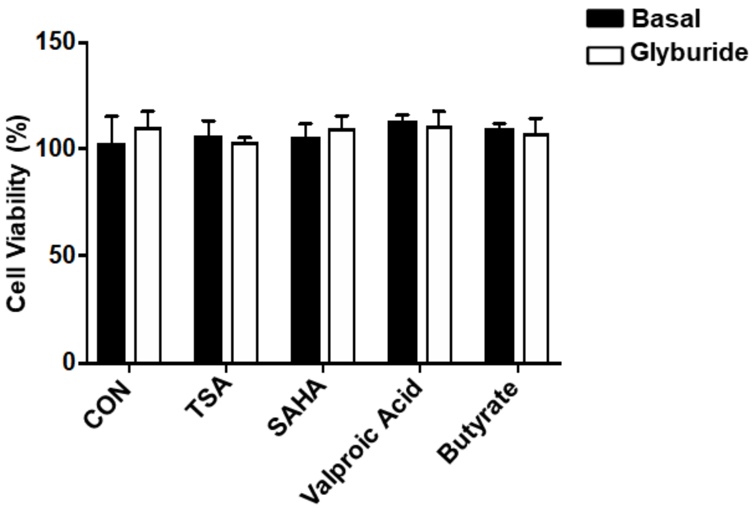


**Figure S1:** HDAC inhibitors induced APE1/Ref-1 secretion in HEK293T cells. (A) Effect of HDAC inhibitors on APE1/Ref-1 secretion in HEK293T cells. Cells (2 x 105 cells) were pretreated with trichostatin A (TSA, 1μM), SAHA (0.5 μM), valproic acid (1 mM), butyrate (0.5 mM) for 1h, then glyburide (10 μM), an ABCA1 transporter inhibitor was treated for 1 h. The total amount of secreted APE1/Ref-1 in cell-free supernatant was analyzed by ELISA as described in Material and Methods. Column mean (n = 4); bars, SE.* $p < 0.05$, significantly different compared with control. [#] $p < 0.05$, significantly different compared with Basal and glyburide group by one-way ANOVA followed by Bonferroni's multiple comparison test. (B) Effect of glyburide and HDAC inhibitors on the viability of HEK293T cells were determined using a RealTime-GloTM MT luminescent kit. Note: HDAC inhibitors [TSA (1μM), SAHA (0.5 μM), valproic acid (1 mM), butyrate (0.5 mM)] used did not significantly affect cell viability.
